# Vomiting in pregnancy is associated with a higher risk of low birth weight: a cohort study

**DOI:** 10.1186/s12884-018-1786-1

**Published:** 2018-05-04

**Authors:** Clive J. Petry, Ken K. Ong, Kathryn Beardsall, Ieuan A. Hughes, Carlo L. Acerini, David B. Dunger

**Affiliations:** 1 Department of Paediatrics, University of Cambridge, Box 116, Cambridge Biomedical Campus, Hills Road, Cambridge, CB2 0QQ UK; 2 Medical Research Council Epidemiology Unit, University of Cambridge, Cambridge, CB2 0QQ UK; 3 The Institute of Metabolic Science, University of Cambridge, Cambridge, CB2 0QQ UK

**Keywords:** Hyperemesis gravidarum, Nausea, Anti-emetic, Small for gestational age

## Abstract

**Background:**

Low birth weight has important short- and long-term health implications. Previously it has been shown that pregnancies affected by hyperemesis gravidarum in the mother are at higher risk of having low birth weight offspring. In this study we tested whether such risks are also evident with less severe nausea and vomiting in pregnancy.

**Methods:**

One thousand two hundred thirty-eight women in the prospective Cambridge Baby Growth Study filled in pregnancy questionnaires which included questions relating to adverse effects of pregnancy and drugs taken during that time. Ordinal logistic regression models, adjusted for parity, ethnicity, marital and smoking status were used to relate the risk of giving birth to low birth weight (< 2.5 kg) babies to nausea and/or vomiting in pregnancy that were not treated with anti-emetics and did not report suffering from hyperemesis gravidarum.

**Results:**

Only three women in the cohort reported having had hyperemesis gravidarum although a further 17 women reported taking anti-emetics during pregnancy. Of those 1218 women who did not take anti-emetics 286 (23.5%) did not experience nausea or vomiting, 467 (38.3%) experienced nausea but not vomiting and 465 experienced vomiting (38.2%). Vomiting during pregnancy was associated with higher risk of having a low birth weight baby (odds ratio 3.5 (1.2, 10.8), *p* = 0.03). The risk associated with vomiting was found in the first (*p* = 0.01) and second (p = 0.01) trimesters but not the third (*p* = 1.0). The higher risk was not evident in those women who only experienced nausea (odds ratio 1.0 (0.3, 4.0), p = 1.0).

**Conclusions:**

Vomiting in early pregnancy, even when not perceived to be sufficiently severe to merit treatment, is associated with a higher risk of delivering a low birth weight baby. Early pregnancy vomiting might therefore be usable as a marker of higher risk of low birth weight in pregnancy. This may be of benefit in situations where routine ultrasound is not available to distinguish prematurity from fetal growth restriction, so low birth weight is used as an alternative.

**Electronic supplementary material:**

The online version of this article (10.1186/s12884-018-1786-1) contains supplementary material, which is available to authorized users.

## Background

Low birth weight (LBW) leads to a higher risk of perinatal mortality and morbidity, including impaired growth and cognitive development [1]. More long-term complications include higher risks for high blood pressure [2] and cardiovascular disease [3], impaired glucose tolerance and type 2 diabetes [4], early age at menarche [5] and menopause [6], and reduced bone mineral density [7] and osteoporosis [8]. LBW can relate to one or both of premature birth and fetal growth restriction, or being constitutionally small, and risk of LBW can be related to such factors as ethnic differences, multiple birth pregnancies, maternal age at birth, fetal environmental factors such as exposure to alcohol, smoking or illicit drugs, maternal nutrition during pregnancy, poor socioeconomic status [9] and genetic defects [10]. Another risk factor appears to be hyperemesis gravidarum [11–13], a severe form of nausea and vomiting in pregnancy that can lead to maternal dehydration and weight loss. Treatment of severe nausea and vomiting in pregnancy with anti-emetics may even be associated with a reduction in the prevalence of LBW [14, 15], although such findings are by no means universal [16–18].

Whilst the association between hyperemesis gravidarum and higher risk of LBW is reasonably well established, what is not so clear is whether potentially less severe nausea and/or vomiting in pregnancy is also associated with the risk of delivering LBW babies. The only recent related evidence suggests that it may be associated with being small for gestational age (SGA) due to fetal growth restriction, one of the main reasons for a baby having a LBW [19]. This study was therefore designed to test the hypothesis that nausea and vomiting in pregnancy, of insufficient severity to require treatment, is associated with the risk of delivering a LBW baby. To do this we used data collected for the Cambridge Baby Growth Study.

## Methods

### Cohort

The prospective and longitudinal Cambridge Baby Growth Study recruited 2229 mothers (and their partners and offspring) attending ultrasound clinics during early pregnancy at the Rosie Maternity Hospital, Cambridge, United Kingdom, between 2001 and 2009 [20]. All mothers were over 16 years of age. Birth weights of their babies, their sex and their gestational age at birth were extracted from hospital notes, having been recorded there by midwives. LBW was defined as a birth weight of less than 2.5 kg. SGA was classified as being in the lowest tenth percentile for gestational age. Prematurity was defined as being born prior to week 37 of gestation. Categorisation according to whether or not the participants developed gestational diabetes [21] or gestational hypertension [22] has been described previously. In this cohort, 96.9% of the offspring were of white ethnicity, 0.8% were of mixed race, 0.6% were black (African or Caribbean), 0.8% were East-Asian, and 0.9% were Indo-Asian.

Each of the study participants was given a printed questionnaire at recruitment with questions to answer and return once the pregnancy was completed [23]. They were encouraged to fill it in as the pregnancy progressed. The questionnaires included boxes to tick if the participants had experienced nausea or had vomited during pregnancy. If either of these boxes were ticked there were further boxes to fill in concerning the timing (i.e. week(s) of pregnancy) when the nausea or vomiting were experienced. A further question asked “Have you taken any medicine during this pregnancy?” Those women who responded in the affirmative were then asked to complete a table with the following headings: “Name”, “Disease”, “Daily Dose”, “No. of Days” and “Gestational Week(s)”. This means that nausea and vomiting prior to attending the booking clinic would have had to have been recalled over a maximum period of several weeks whereas nausea and vomiting subsequent to that could be recorded as the pregnancy progressed (requiring recollection over a much shorter period of time). A total of 1238 women (54.6%) filled in the questionnaires; those that did not were excluded from the present analysis. For 598 of the pregnancies where the mother failed to return a filled-in questionnaire the baby’s birth weight was also missing. The birth weights of the remaining babies, adjusted for pre-pregnancy maternal BMI, gestational age at birth, parity and sex, were not different between those that completed the questionnaire and those that did not (filled in questionnaire 3.482 (3.456, 3.508) kg v. did not fill in questionnaire 3.403 (3.310, 3.497) kg, *p* = 0.1), although the prevalence of LBW in the offspring of the women that returned the questionnaires was lower (filled in questionnaire 37/1218 v. did not fill in questionnaire 27/431, *p* = 5 × 10^− 3^). Of those women that filled in their questionnaires only 3 reported that they had hyperemesis gravidarum and a further 17 were treated with anti-emetics: cyclizine (7), promethazine (5), prochlorperazine (4), metoclopramide (2), domperidone (2), prednisolone (2), chlorphenamine (1), ondansetron (1), chlorpromazine (1) and unknown (1). These 20 women were excluded from this specific analysis in order to test only those women who had a potentially milder phenotype. The self-reported timings of exposure to nausea and/or vomiting were divided into trimesters (first trimester being up to gestational week 12, second trimester being weeks 13 to 27 and third trimester being from week 28 onwards).

### Categorisation and calculations

We categorised each of the women into one of three different groups: those who reported neither nausea nor vomiting (*n* = 286), those who reported nausea but not vomiting (*n* = 467) and those who reported vomiting (*n* = 465, 432 of whom reported nausea and vomiting and 33 who reported vomiting without nausea). Those that reported vomiting without nausea had no evidence of concurrent urinary or chest infections, or evidence of the vomiting occurring just in the final trimester of pregnancy. The body mass indexes (BMI) were calculated dividing the maternal weights prior to pregnancy by their heights squared.

### Statistical analysis

Associations with LBW were adjusted for the following confounders: parity, marital and smoking statuses, and ethnicity [24]. Associations between nausea and vomiting and quantitative continuous variables (such as BMI and maternal age) were tested using linear regression models (adjusted for confounders where necessary). Associations with dichotomous variables (such as LBW) were tested using ordinal logistic regression (adjusted for confounders where necessary), the χ^2^-test or Fisher’s exact test as appropriate. Unless otherwise stated all other data are presented as means (95% confidence intervals (CI)). Statistical analyses were performed using Stata 13 (StataCorp LP, College Station, Texas, U.S.A.). *P* < 0.05 was considered statistically significant throughout.

## Results

### Maternal clinical characteristics

Table 1 shows the clinical characteristics of the different groups tested. Those 465 women who experienced vomiting during pregnancy (432 of whom also reported nausea) tended to be younger (*p* = 1.5 × 10^− 3^) and slightly heavier/more obese (pre-pregnancy weight *p* = 0.02, pre-pregnancy BMI *p* = 0.01) than those women who did not experience nausea or vomiting in pregnancy. These differences in clinical characteristics from those women who did not experience nausea or vomiting, were not evident in those women who experienced nausea but not vomiting.

**Table 1:** Characteristics of the pregnant women in the Cambridge Baby Growth Study who returned their questionnaires categorised according to whether or not they experienced nausea or vomiting in pregnancy

Characteristic	No Nausea or Vomiting	Nausea alone	Vomiting
n	286	467	465
Age (years)	34.2 (33.7, 34.6)	33.6 (33.4, 33.9)	33.1*** (32.7, 33.4)
Height (m)	166.1 (165.3, 166.8)	165.9 (165.5, 166.3)	165.7 (165.1, 166.4)
Pre-pregnancy weight (kg)	65.0 (63.6, 66.4)	66.1 (65.3, 66.9)	67.1* (66.0, 68.3)
Pre-pregnancy BMI (kg/m^2^)	23.6 (23.1, 24.1)	24.0 (23.7, 24.3)	24.5* (24.0, 24.9)
Weight gain during pregnancy (kg)	7.9 (7.1, 8.7)	8.2 (7.7, 8.6)	8.4 (7.7, 9.1)
Smoked during pregnancy (%)	4.2	1.9	4.6
Developed gestational diabetes (%)	10.6	9.6	9.6
Developed gestational hypertension (%)	6.9	4.2	7.2

*P < 0.05, **P < 0.01, ***P < 0.001 compared with the no nausea or vomiting group. Data are mean (95% CI). None of these women were treated with anti-emetics during pregnancy. Nausea alone refers to those women who experienced nausea in pregnancy but not vomiting. Vomiting refers to those women who experienced vomiting during pregnancy, independently of whether or not they also experienced nausea

### Associations between maternal exposure to nausea and/or vomiting in pregnancy and birth characteristics of the baby

The delivery of LBW babies was more common in those women who experienced vomiting in pregnancy (when adjusted for confounders) than in those women who did not experience vomiting or nausea (*p* = 0.03; Table 2). This relationship was still evident after further adjustment was made for maternal BMI prior to pregnancy (OR 3.1 (1.0, 9.7), *p* = 0.048). In contrast there was no higher risk of LBW associated with experiencing nausea but not vomiting in pregnancy (*p* = 1.0).

**Table 2:** Birth related factors of babies of women in the Cambridge Baby Growth Study who returned their questionnaires, categorised according to whether they experienced nausea or vomiting in pregnancy

Birth-related Factor	No Nausea or Vomiting	Nausea alone	Vomiting
n	286	467	465
Parity (n)	1.7 (1.6-1.8)	1.8 (1.7-1.8)	1.8* (1.8-1.9)
Birth weight (kg)	3.512 (3.459, 3.565)	3.504 (3.474, 3.534)	3.496 (3.453, 3.540)
Adjusted birth weight (kg)	3.494 (3.449, 3.539)	3.499 (3.474, 3.525)	3.505 (3.468, 3.541)
LBW (n/total (%))	6/277 (2.2%)	9/461 (2.0%)	21/460* (4.6%)
LBW (OR)	Ref.	1.0 (0.3-4.0)	3.5* (1.2-10.8)
LBW (OR) *first trimester nausea or vomiting*		2.1 (0.8-5.7)	4.3* (1.4-13.2)
LBW (OR) *second trimester nausea or vomiting*		4.2* (1.7-10.1)	4.4* (1.4-13.9)
LBW (OR) *third trimester nausea or vomiting*		1.5 (0.3-6.7)	0.8 (0.1-7.0)
Gestational age at birth (weeks)	39.9 (39.8, 40.1)	39.9 (39.8, 40.0)	39.8 (39.7, 39.9)
Prematurity (n (%))	4 (1.4)	7 (1.5)	11 (2.3)
SGA (n (%))	11 (3.9)	16 (3.5)	25 (5.2)
Sex of the baby (n (%) male)	161 (56.9%)	236 (51.1%)	225 (49.9%)*

*P < 0.05, **P < 0.01, ***P < 0.001 compared with the no nausea or vomiting group. None of these women were treated with anti-emetics during pregnancy. Nausea alone refers to those women who experienced nausea in pregnancy but not vomiting. Vomiting refers to those women who experienced vomiting during pregnancy, independently of whether or not they also experienced nausea. Birth weight is presented both unadjusted and adjusted for pre-pregnancy maternal BMI, gestational age at birth, parity, multi-fetal pregnancy and sex. LBW OR was adjusted for parity, marital status, ethnicity and maternal smoking status. Data are mean (95% CI) or number (%)

**Fig. 1:**
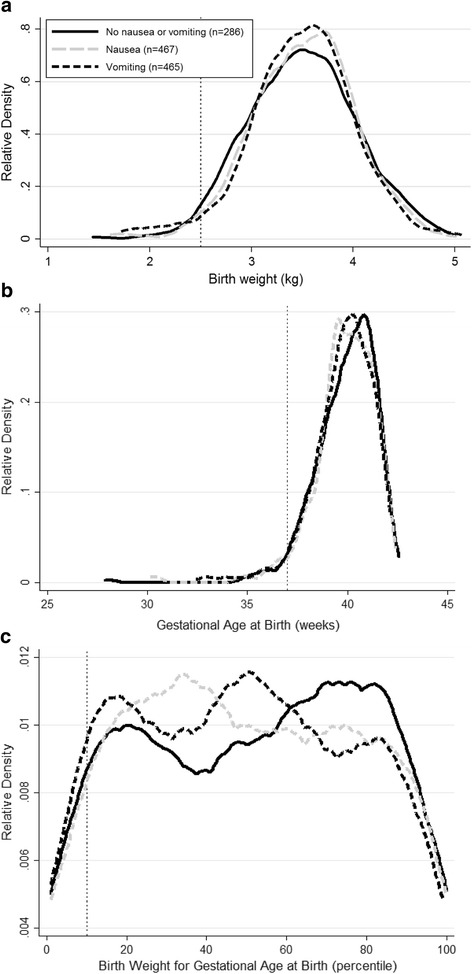
Kernel density estimation plots of **a** unadjusted birth weight distributions, **b** gestational age at birth and **c** birth weight adjusted for gestational age at birth percentiles in the Cambridge Baby Growth Study in babies whose mothers were not treated with anti-emetics in pregnancy. The lines are plotted according to the mother’s exposure to nausea or vomiting in pregnancy. The cut off for LBW is shown by the dotted line at 2.5 kg in (**a**), that for prematurity is shown by the dotted line at 37 weeks in (**b**) and that for SGA is shown by the dotted line at the tenth percentile of birth weight adjusted for gestational age at birth in (**c**). Nausea refers to those women who experienced nausea but not vomiting during pregnancy. Vomiting refers to those women who experienced vomiting during pregnancy, independently of whether or not they also experienced nausea

Table 2 shows other birth characteristics of women according to their experience of nausea and vomiting in pregnancy. Despite the differences in risk of LBW, there was no apparent difference in mean birth weight, gestational age at birth or prevalence of prematurity or SGA. Similarly there was no apparent difference in the birth weight adjusted for pre-pregnancy maternal BMI, gestational age at birth, sex and parity. However there was a slightly higher proportion of female babies born to mothers who experienced vomiting during pregnancy. Figure 1 shows Kernel density estimation plots for unadjusted birth weight, gestational age at birth and birth weight for gestational age according to maternal exposure to nausea and vomiting in pregnancy. Small differences in the distributions of these were evident in each plot.

### Associations between exposure to vomiting in different trimesters of pregnancy and birth characteristics of the baby

The higher prevalence of giving birth to LBW babies in women who experienced vomiting was evident in those who experienced it in the first (OR 4.3 (1.4, 13.2), *p* = 0.01, *n* = 266 vomiting and *n* = 204 no nausea or vomiting) or second (OR 4.4 (1.4, 13.9), p = 0.01, *n* = 185 vomiting and n = 204 no nausea or vomiting) trimester of pregnancy. However it was not evident in those who experienced it in the third trimester (OR 1.0 (1.0, 1.0), *n* = 36 vomiting and 204 no nausea or vomiting, *p* = 1.0).

## Discussion

Vomiting in pregnancy, not treated with anti-emetics, is associated with a higher risk of giving birth to LBW babies in this study. This is consistent with reported associations between LBW (or related phenotypes such as SGA) and hyperemesis gravidarum [11–13, 25–28], although such associations are not universal findings [29–32]. Nausea and vomiting with no reference to hyperemesis gravidarum has also been associated with a higher risk of LBW [33] and decreased birth weight [34] in some other studies, although no difference in risk was reported in others [19, 35–37]. Although a systematic review [38] reported a lower risk of LBW in association with nausea and vomiting in pregnancy the studies that it was based looked at anti-emetic use to categories study participants [14, 15]. This is therefore very different to our own study where we specifically excluded women who took anti-emetics in case these drugs affected pathways involved in regulating LBW risk [39]. Similarly the very large Norwegian Mother and Child Cohort Study [40] found reduced population rates of LBW in association with nausea and vomiting in pregnancy, but did so without specifically excluding those women who took anti-emetics in pregnancy. Our study therefore presents results a slightly different population to those examined in other investigations, and one where we specifically investigated a potentially milder negative aspect of pregnancy than hyperemesis gravidarum.

This higher risk for LBW associated with vomiting, and nausea but just in the second trimester, appeared to be in the first two trimesters of pregnancy with an apparent lack of risk in the third trimester. Studies are ongoing to try and discover whether or not nausea and vomiting in pregnancy and resulting associations are genetically mediated [41, 42]. With an overall odds ratio of 3.5 for LBW in our population, vomiting in early pregnancy may be a marker of risk for LBW that is useful for its prediction in conjunction with other risk factors. Where routine ultrasound is available small babies tend to be assessed as either SGA (possibly due to fetal growth restriction) and/or premature rather than LBW, but the link with vomiting may be useful in areas where such scans are not available and LBW is used to encompass them both. Nausea and vomiting in pregnancy are thought to be protective towards the embryo/fetus in terms of reducing exposure to food borne harmful substances such as infective microorganisms [43], and they can lead to changes in the maternal dietary intake [44]. There is evidence that this can lead to positive effects on the fetus such as decreased rates of miscarriage and congenital malformations [45]. Whilst potentially being an advantage in a mild form, in excess it is possible that this vomiting might reduce nutrient delivery to the fetus leading to the greater risk of LBW [46]. The fact that in our population as a whole there was no apparent decrease in mean birth weight despite the higher prevalence of LBW suggests that whilst there is a negative impact of vomiting on birth weight for some babies, in other babies a protective advantage may be evident [43]. The Kernel density estimation plot for birth weight in our population (Fig. 1(a)) would appear to be consistent with this suggestion (as birth weight density around the mean appeared to be higher in those women affected by vomiting).

LBW often relates to the baby undergoing fetal growth restriction and/or being born prematurely. Given the increase in birth weight usually observed in male babies, in general there is also a slightly increased risk of LBW in female babies compared to males [9]. In our population despite the association between vomiting and LBW, we did not find further significant independent associations between exposure to vomiting in pregnancy and the prevalence of SGA (a group that may have been enriched with babies who underwent fetal growth restriction) or premature births or the gestational age at birth. Looking at the Kernel density estimation plots for birth weight in our population below the cut off for LBW the line for women who experienced vomiting was clearly a little higher than those representing the other groups (Fig. 1(a)). The differences between the groups below the cut offs in the gestational ages at birth (to assess the densities related to prematurity) and the birth weights for gestational age (to assess the densities related to SGA) plots were smaller though (Fig. 1 (b) and (c)). Further studies are required to validate these findings in other cohorts, especially those that are better able to distinguish whether the LBW relates more to SGA (and therefore probably fetal growth restriction), prematurity or a mix of the two.

Whilst we could not ascribe the increased risk of LBW to fetal growth restriction or premature birth we did note a slight excess of pregnant women carrying female babies. This excess has previously been observed with hyperemesis gravidarum [47]. We recently reported that serum GDF-15 concentrations around week 15 of pregnancy were higher in those women reporting vomiting in the second trimester of pregnancy in the Cambridge Baby Growth Study [48], a hormone that may stimulate vomiting in pregnancy [49]. Interestingly we have also observed an increased concentration of week 15 serum GDF-15 concentrations in women carrying females compared to those carrying males in our population (Petry et al., unpublished observation), which may explain the slight excess of pregnant women carrying females in the group that experienced vomiting.

The main strengths of this study are its prospective nature and the fact that its design enabled us to study a group of women with a potentially milder, albeit still unpleasant phenotype than has been tested in other published studies. We have therefore uniquely been able to observe that the risk of giving birth to a LBW baby is higher with vomiting. The study’s main limitation is that the nausea and vomiting and the taking of any anti-emetics to treat it were self-reported. The analysis could therefore have been affected by recall bias [50], although given that the women were encouraged to fill in their questionnaires as their pregnancies progressed rather than retrospectively the effects of this may have been limited. Indeed effects of any recall bias were clearly not sufficient to prevent us from discovering our association between increased circulating GDF-15 concentrations and second trimester vomiting [48]. Another limitation of our study may be a slight lack of statistical power to investigate potential effects of confounders*.* However there was clearly sufficient power in our study to test associations between the maternal experience of nausea and vomiting in pregnancy and the risk of delivering LBW babies. The final main limitation may be exclusion of women who took anti-emetics during pregnancy. This is because the threshold for nausea and vomiting where women sought and were then prescribed anti-emetics would have varied from participant to participant, and therefore the exclusion could have been rather self-selecting. However the advantage of doing this is the lack of potential confounding in our study of these drugs affecting pathways involved in regulating fetal growth, a strength of the study.

## Conclusions

This study suggests that vomiting in early pregnancy of insufficient severity to merit self-selected treatment with anti-emetics is associated with a higher risk of a woman giving birth to a LBW baby. It is possible that this vomiting may therefore be a marker of LBW pregnancies. This could be useful in situations where routine ultrasound is not available to distinguish prematurity from being SGA, so LBW is used as an alternative to encompass them both.

## Additional file

Additional file 1: Description of data: the data that was associated with the manuscript entitled “Vomiting in pregnancy is associated with a higher risk of low birth weight: a cohort study” by Petry et al. (https://doi.org/10.1186/s12884-018-1786-1) (XLSX 149 kb)

## Abbreviations

BMI: Body mass index
CI: Confidence interval
LBW: Low birth weight
OR: Odds ratio
SGA: Small for gestational age
